# Exploring male involvement and support in long-acting reversible contraceptive decision-making: a mixed-methods cross-sectional study in Kampala, Uganda

**DOI:** 10.1186/s40834-026-00468-6

**Published:** 2026-08-01

**Authors:** Brian Nyasulu, Nicholas Ngomi, Ronald Arineitwe Kibonire

**Affiliations:** Department of Public Health, Kampala International University, P. O. Box 20000, Kampala, Uganda

**Keywords:** Male involvement, Family planning, LARCs, Reproductive health, Cultural norms

## Abstract

**Background:**

Male involvement in family planning is a critical yet underexplored factor influencing contraceptive uptake and decision-making, particularly for long-acting reversible contraceptives (LARCs). In Kampala, Uganda, socio-cultural norms and misconceptions may limit men’s participation in reproductive health services. This study assessed the extent, determinants, and barriers to male involvement in LARC decision-making among couples.

**Methods:**

A mixed-methods cross-sectional study was conducted between March and September 2025 in Makindye Division and Kisugu Health Centre III. Quantitative data were collected from 362 women using structured questionnaires, while qualitative data were obtained from six focus group discussions (FGDs) with men and women, and five key informant interviews (KIIs) with healthcare providers. Quantitative data were analyzed using SPSS, and qualitative data were thematically analyzed in NVivo. Instruments were pre-tested and assessed for reliability.

**Results:**

While 96% of women reported that their partners discussed contraception with healthcare providers, only 10% accompanied their partners to clinics. Male support for family planning was mainly financial (60%), and joint decision-making on LARCs occurred in 39% of couples. Key determinants of male involvement included marital status, education level, and number of children. Cultural norms and religious beliefs emerged as significant barriers. Qualitative findings highlighted entrenched gender norms, misconceptions about contraceptives, and the perception that reproductive health services are primarily for women. Participants reported limited couple-focused counseling, with only 44% indicating men’s concerns were adequately addressed during consultations.

**Conclusions:**

Male involvement in LARC decision-making in Kampala remains low due to socio-cultural barriers and limited male-friendly services. Strengthening male engagement requires inclusive counseling by trained health workers, community and religious leader involvement, and promotion of male-friendly and couple-centered reproductive health services.

**Supplementary Information:**

The online version contains supplementary material available at https://doi.org/10.1186/s40834-026-00468-6.

## Introduction

Family planning is a critical component of reproductive health, contributing significantly to improved maternal and child health outcomes, reduced unintended pregnancies, and enhanced gender equality (UNFPA, 2021). Despite the well-documented benefits of family planning, male involvement in these programs remains limited, particularly concerning the use of long-acting reversible contraceptive (LARC) methods. Traditionally, family planning initiatives have predominantly targeted women, often overlooking the influential role men play in reproductive decision-making within many societies [1].

In this study, male involvement is conceptualized as a multidimensional construct that encompasses men’s active participation in family planning through communication with partners, joint decision-making on contraceptive use, provision of financial and emotional support, and engagement with health services, such as accompanying partners to clinics [2]. This involvement is further understood as being shaped by socio-cultural norms, gender dynamics, and access to reproductive health information, all of which influence men’s attitudes and behaviors toward family planning. By adopting this comprehensive definition, the study aims to provide a more nuanced understanding of male participation in family planning, particularly in relation to the uptake and support of LARC methods [14]. This gender imbalance hampers the effectiveness of family planning programs and underscores the need to actively engage men as partners in reproductive health.

Globally, healthcare providers are recognized as pivotal actors in promoting male participation in family planning by creating inclusive, male-friendly environments and providing education to dispel myths and misconceptions about contraceptives [12]. Research from a variety of settings shows how crucial provider attitudes, communication abilities, and collaborative decision-making are in encouraging male participation, which improves reproductive health outcomes [9]. Healthcare professionals in Tanzania, for example, expressed pleasure with the rise in male involvement but also pointed out that service delivery is constrained by resource and infrastructure shortages [10].

Data from Uganda’s National Family Planning Costed Implementation Plan (2020/21–2024/25) shows that male participation in family planning is alarmingly low, with a prevalence rate of just 28.4% [17]. Similarly, the 2024 Uganda Bureau of Statistics report indicates that only 38% of married women aged 15 to 49 use any contraceptive method (UBOS [15]).These statistics underscore a critical gap in family planning service delivery and uptake, particularly among men, which undermines national efforts to reduce unintended pregnancies and improve maternal and child health.

Additional obstacles that healthcare workers in Sub-Saharan Africa must overcome include cultural norms that discourage male involvement, a lack of resources, and poor training [18]. To promote male involvement through outreach initiatives and education, providers work to integrate culturally appropriate approaches and partner with community leaders [8]. Male-centered care and community-based programs are the main focus of current efforts in Uganda to raise male awareness and accessibility to family planning services, especially in underserved and rural areas [16]. However, obstacles such enduring gender stereotypes, false information, and restrictions on the health system still prevent males from participating, particularly in urban and peri-urban areas like Kampala [3].

Despite these revelations, there is still a great deal of unanswered questions about the precise role that healthcare professionals play in encouraging male involvement in LARC usage, especially in Ugandan urban slum areas. Male engagement is typically aggregated in research without a detailed analysis of views or support for LARC procedures [6]. Furthermore, in these settings, provider biases, insufficient infrastructure, and cultural challenges have not yet been thoroughly investigated [3]. To improve male participation in family planning and reproductive health outcomes, it is essential to address these gaps and develop targeted interventions that consider the unique socioeconomic and cultural dynamics of urban populations.

## Methodology

This study employed a concurrent mixed-methods cross-sectional design to examine male involvement in long-acting reversible contraceptive (LARC) decision-making among couples in Kampala, Uganda. Data were collected between 24 March 2025 and 30 September 2025 in Makindye Division and Kisugu Health Centre III, allowing the quantitative and qualitative components to be conducted simultaneously and integrated during analysis for a comprehensive understanding of male participation.

For the quantitative component, 362 women aged 18 years and above attending family planning services were recruited using systematic random sampling. Eligible participants were women in marital or cohabiting relationships who had lived in the study area for at least six months; those who were severely ill or declined participation were excluded. Qualitative data were obtained through six focus group discussions (FGDs) with men and women, semi-structured interviews with women and their male partners, and five key informant interviews (KIIs) with healthcare providers involved in family planning. Participants for FGDs and interviews were purposively selected to ensure representation across age, marital status, and education levels.

The structured questionnaire and qualitative interview guides were developed specifically for this study, informed by existing literature on male involvement in family planning and LARC use. The instruments captured socio-demographic characteristics, knowledge and awareness of LARC methods, patterns of male involvement, communication between partners, and perceived barriers to male participation. To ensure clarity, relevance, and cultural appropriateness, the tools were pre-tested among a similar population at a neighboring health facility not included in the study. Feedback from the pre-test was used to refine question wording, sequence, and response options. Reliability of the quantitative questionnaire was assessed using Cronbach’s alpha, which indicated acceptable internal consistency (α ≥ 0.7), while content validity was ensured through expert review by public health specialists and reproductive health practitioners.

Qualitative data were analyzed thematically using NVivo software, following an inductive coding approach to allow themes to emerge naturally from the data. Two independent researchers coded transcripts, identifying meaningful units of text that were grouped into broader categories and overarching themes. Discrepancies in coding were resolved through consensus, with a third reviewer consulted where necessary to ensure reliability and trustworthiness.

Quantitative data were analyzed using SPSS, with descriptive statistics summarizing participant characteristics and key variables. Chi-square tests assessed associations between male involvement and selected covariates, and variables with significant associations were further included in a multivariable logistic regression model to identify independent predictors of high male involvement in LARC decision-making, adjusting for marital status, education, parity, and other relevant factors.

Ethical approval was obtained from the Kampala International University Research Ethics Committee (KIU-REC) (Approval No. KIU-2024-563) and the Uganda National Council for Science and Technology (UNCST) (Approval No. HS5778ES). All participants provided written informed consent, and confidentiality and cultural sensitivity were maintained throughout the study.

## Results

**Fig. 1:**
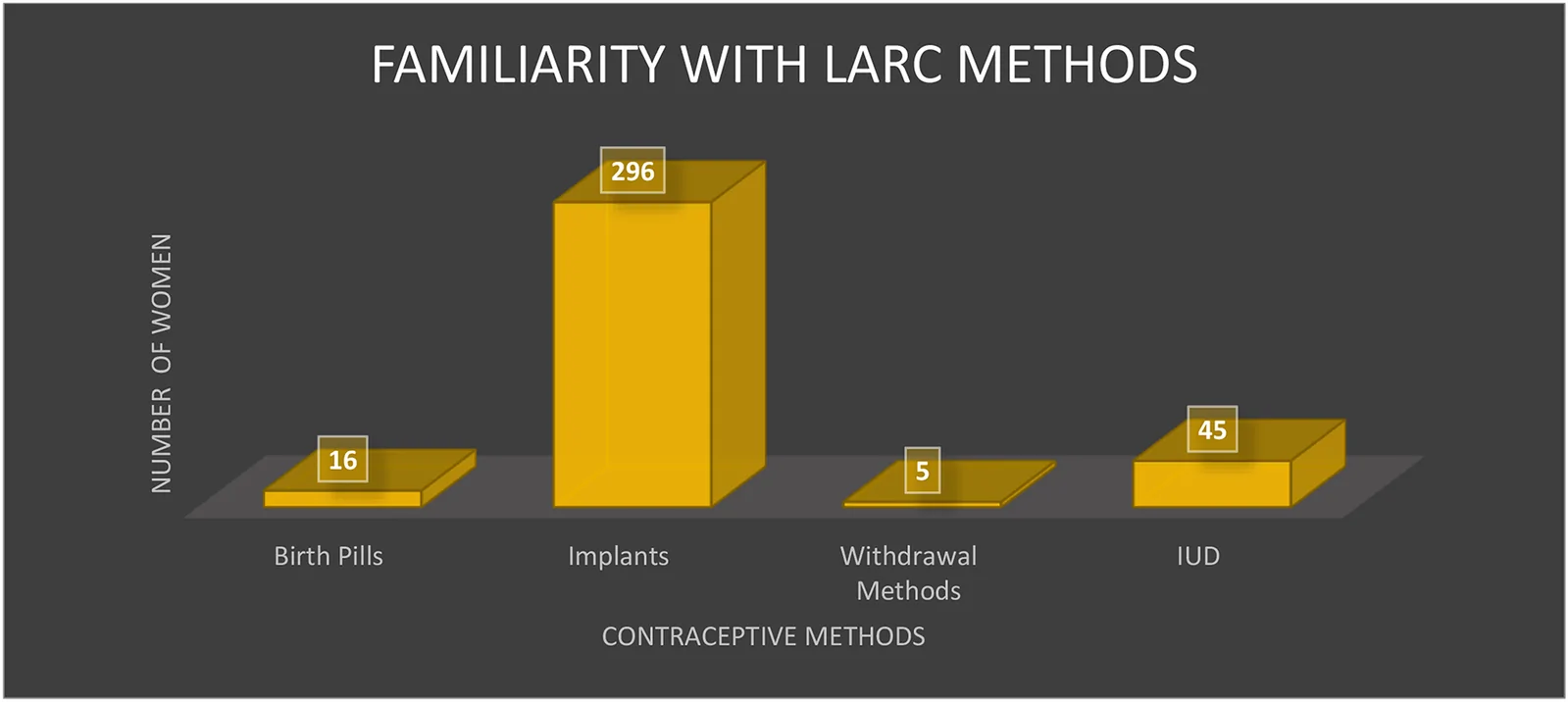
Familiarity with LARC methods

The findings in Fig. 1 below shows that among women respondents, implants were the most recognized long-acting reversible contraceptive (LARC) method (81.8%), followed by intrauterine devices (IUDs) (12.4%). A smaller proportion of women were familiar with birth control pills (4.4%) and natural methods such as withdrawal (1.4%), as shown in (Fig. 1) below. This pattern indicates a high level of awareness of implants compared to other methods, while knowledge of alternative contraceptive options remains relatively low. Such variation in awareness may influence contraceptive choices and the extent of male involvement in decision-making regarding family planning.

**Table 1:** Chi-Square analysis on the extent of male involvement in decision-making about long-acting reversible contraceptives (LARC) among couples in Kampala

Statement/Question	Response	Freq (%)	χ²	*p*-value
Do you believe that contraceptive use can improve a couple’s relationship?	Strongly disagree	3 (0.8%)	41.131	0.001*
	Disagree	20 (5.5%)		
	Neutral	70 (19.3%)		
	Agree	192 (53.0%)		
	Strongly agree	77 (21.3%)		
	**Total**	**362 (100%)**		
Do you think men should be involved in contraceptive decisions?	Strongly disagree	2 (0.6%)	49.423	0.004*
	Disagree	13 (3.6%)		
	Neutral	49 (13.5%)		
	Agree	216 (59.7%)		
	Strongly agree	82 (22.7%)		
	**Total**	**362 (100%)**		
Do you believe that using LARC is important for family planning?	Strongly disagree	5 (1.4%)	36.675	0.002*
	Disagree	17 (4.7%)		
	Neutral	69 (19.1%)		
	Agree	210 (58.0%)		
	Strongly agree	61 (16.9%)		
	**Total**	**362 (100%)**		
Have you heard about LARC methods?	Yes	357 (98.6%)	231.5	0.001*
	No	5 (1.4%)		
	**Total**	**362 (100%)**		
How does your husband support you in your choice of contraceptive method?	Accompany her to the clinic	35 (9.7%)	17.246	0.141
	Provide financial support and don’t accompany her	218 (60.2%)		
	Offer emotional support	73 (20.2%)		
	Do not support	36 (9.9%)		
	**Total**	**362 (100%)**		
How should men be involved in LARCs?	Not involved at all	4 (1.1%)	94.981	0.003*
	Minimally involved	20 (5.5%)		
	Neutral	70 (19.3%)		
	Involved	198 (54.8%)		
	Very involved	70 (19.3%)		
	**Total**	**362 (100%)**		
Husband’s participation in choosing a LARC?	Yes	140 (38.7%)	2.776	0.596
	No	222 (61.3%)		
	**Total**	**362(100%)**		
How often do you discuss LARC options with your partner?	Always	9 (2.5%)	78.658	0.002*
	Often	54 (14.9%)		
	Sometimes	153 (42.3%)		
	Rarely	121 (33.5%)		
	Never	25 (6.9%)		
	**Total**	**362 (100%)**		

The study looked at how men are involved in family planning, especially in the use of long-acting reversible contraceptives (LARCs), among 362 participants in Kampala, Uganda in the Table 1 above. Most respondents (74.3%) agreed that using contraceptives can improve a couple’s relationship (*χ² = 41.131*, *p* = 0.002), and 82.4% believed men should be involved in making contraceptive decisions (*χ² = 49.423*, *p* < 0.001). About 74.9% said LARCs are important for family planning (*χ² = 36.675*, *p* = 0.002).

Awareness of LARC methods was very high, with 98.6% having heard of them (*χ² = 231.500*, *p* < 0.001). However, only 38.7% of couples had chosen a method together, which was not statistically significant (*χ² = 2.776*, *p* = 0.596).

When it came to support, 60.2% of men said their partners gave financial help but did not go with them to clinics. Only 9.7% said their partners accompanied them, which also was not statistically significant (*χ² = 17.246*, *p* = 0.141).

Even though 74.1% believed men should be involved in family planning (*χ² = 94.981*, *p* < 0.003), only 17.4% said they often or always talked about LARC options with their partners (*χ² = 78.658*, *p* < 0.001). This shows that while many support male involvement, actual participation and communication remain low.

**Fig. 2:**
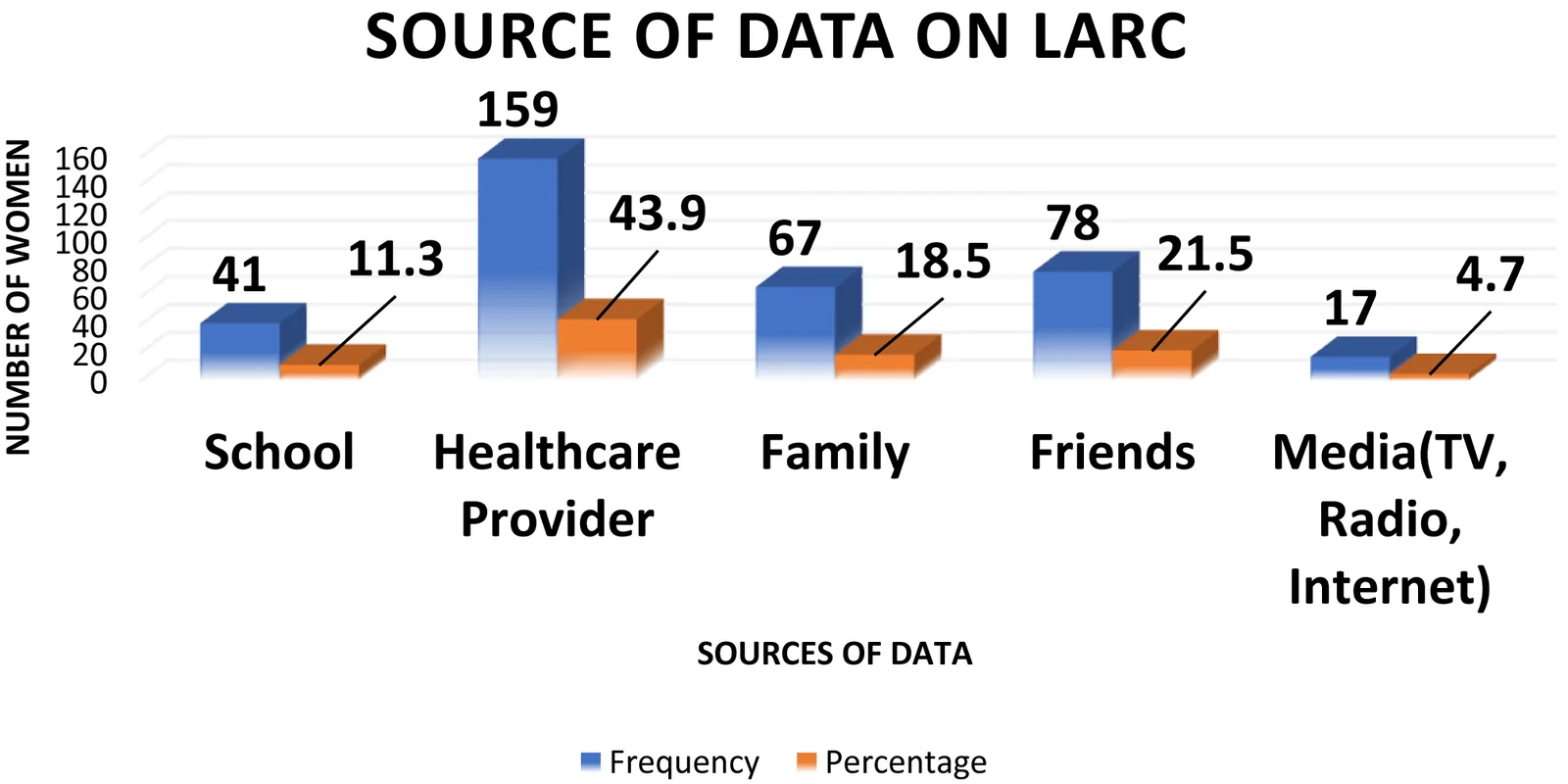
Source of data on LARC. Source: Field Survey 2025

The Fig. 2 below shows Female respondents reported learning about long-acting reversible contraceptive (LARC) methods from various sources. The majority (43.9%) said healthcare providers were their main source of information. Family members (21.5%) and friends (18.5%) also played key roles. Smaller numbers learned about LARCs through school (11.3%) or media platforms like TV, radio, and the internet (4.7%). These findings highlight the diverse channels through which LARC information is shared, which can influence women’s choices and the level of male involvement in family planning decisions, as shown in (Fig. 2) below.

**Table 2:** Chi-Square on factors and barriers influencing male involvement in long-acting reversible contraceptives (LARC) use and decision-making in Kampala, Uganda

Variable	Response	Frequency (%)			χ²	*p*-value
Marital Status	Single		22 (6.1%)	35.627		0.001*
	Married		312 (86.2%)			
	Cohabiting		16 (4.4%)			
	Divorced/Separated		9 (2.5%)			
	Widowed		3 (0.8%)			
	**Total**		**362 (100%)**			
Education Level	No formal education		8 (2.2%)	44.289		0.002*
	Primary school		57 (15.7%)			
	Secondary school		157 (43.4%)			
	Diploma/Certificate		109 (30.1%)			
	Bachelor’s degree		24 (6.6%)			
	Master’s degree or higher		7 (1.9%)			
	**Total**		**362 (100%)**			
Number of Children	None		19 (5.2%)	38.428		0.004*
	One to Two		140 (38.7%)			
	Three to Four		174 (48.1%)			
	Five to Six		28 (7.7%)			
	Seven or More		1 (0.3%)			
	**Total**		**362 (100%)**			
What do you think prevents men from being more involved in LARC?	Lack of knowledge		12 (3.3%)	21.053		0.636
	Cultural beliefs		31 (8.6%)			
	Religious beliefs		76 (21.1%)			
	Fear of side effects		129 (35.7%)			
	Partner’s preference		108 (30.0%)			
	No barriers		4 (1.1%)			
	**Total**		**361 (100%)**			
Cultural norms affect your involvement in LARC?	Strongly disagree		6 (1.7%)	42.813		0.003*
	Disagree		15 (4.1%)			
	Neutral		45 (12.4%)			
	Agree		264 (73.0%)			
	Strongly agree		32 (8.8%)			
	**Total**		**362 (100%)**			
Do religious beliefs influence your decisions about LARC	Strongly disagree		9 (2.5%)	29.402		0.021*
	Disagree		50 (13.8%)			
	Neutral		135 (37.2%)			
	Agree		146 (40.3%)			
	Strongly agree		22 (6.1%)			
	**Total**		**362 (100%)**			

The Table 2 above examined key socio-demographic and belief-related factors influencing male involvement in the use and decision-making around long-acting reversible contraceptives (LARCs) among 362 respondents in Kampala, Uganda.

Marital status was significantly associated with male involvement (χ² = 35.627, *p* = 0.001), with the majority of respondents being married (86.2%), followed by single (6.1%) and cohabiting individuals (4.4%).

Education level also showed a significant association (χ² = 44.289, *p* = 0.002), with most participants having secondary (43.4%) or diploma/certificate education (30.1%).

The number of children was another significant factor (χ² = 38.428, *p* = 0.004), where most respondents had between three to four children (48.1%) or one to two children (38.7%).

Regarding barriers to male involvement in LARCs, fear of side effects (35.7%) and partner preference (30%) were the most cited reasons, followed by religious beliefs (21.1%) and cultural beliefs (8.6%). However, this variable was not statistically significant (χ² = 21.053, *p* = 0.636).

Cultural norms had a strong influence on involvement, with 73% agreeing and 8.8% strongly agreeing, and this was statistically significant (χ² = 42.813, *p* < 0.001).

Similarly, religious beliefs were significantly associated (χ² = 29.402, *p* = 0.021), with 40.3% agreeing and 6.1% strongly agreeing that religion influenced their decisions about LARCs.

Qualitative findings from focus group discussions reinforced these quantitative results. Married men in Kampala shared diverse perspectives on their roles in family planning. One participant in his 30s expressed a sense of responsibility in reproductive decision-making, stating.

In my marriage, I feel responsible for every decision that concerns our family, especially the number of children we plan to have. My wife and I often talk about family planning, and we both agreed to go for a long-term method after our third child. (FGD, Woman 1)

Another participant, a secondary school teacher aged 36–40 years with a diploma in education, highlighted the influence of awareness and education.

I learned about LARC methods during a health outreach session at our local center. Before that, I used to think family planning was just a woman’s issue. But now I know it’s something we should decide on together. Education helped me understand how important this is, not just for her health but for our future as a family. (FGD, Man 2)

Participants consistently emphasized that education, awareness, and financial stability promote joint decision-making, while misinformation, cultural taboos, and fear of stigma remain barriers. Many men expressed the need for accurate information directly from healthcare providers rather than relying on second-hand sources. Strengthening male-focused health education and integrating men into family planning discussions at the facility and community level could therefore enhance shared decision-making and increase support for LARC use among couples.

**Table 3:** Chi-Square analysis on the role of healthcare providers in promoting male involvement in family planning, especially long-acting reversible contraceptives

Statement/Question	Response	Freq (%)	χ²	*p*-value
Do healthcare providers adequately address men’s concerns about LARC?	Strongly disagree	18 (5.0%)	17.663	0.344
	Disagree	86 (23.8%)		
	Neutral	81 (22.4%)		
	Agree	160 (44.2%)		
	Strongly agree	17 (4.7%)		
	**Total**	**362 (100%)**		
Have you ever discussed contraception with a healthcare provider?	Yes	349 (96.4%)	31.366	0.002*
	No	13 (3.6%)		
	**Total**	**362 (100%)**		
Do you think that more education and outreach from healthcare providers would encourage male involvement in family planning?	Disagree	7 (6.7%)	12.309	0.421
	Neutral	3 (2.9%)		
	Agree	37 (35.2%)		
	Strongly agree	58 (55.2%)		
	**Total**	**105 (100%)**		
Have healthcare providers ever directly involved you in family planning discussions with your partner?	Yes	82 (22.7%)	6.294	0.178
	No	280 (77.3%)		
	**Total**	**362 (100%)**		
Do you think healthcare providers adequately address men’s concerns about LARC?	Strongly disagree	2 (12.5%)	17.663	0.344
	Disagree	3 (18.8%)		
	Neutral	21 (13.8%)		
	Agree	53 (34.9%)		
	Strongly agree	3 (19.0%)		
	**Total**	**105 (100%)**		

The quantitative and qualitative findings from this study are strongly complementary and together provide a deeper understanding of male involvement in long-acting reversible contraceptive (LARC) decision-making among couples in Kampala in the Table 3 above.

The qualitative findings were strengthened through the identification of three major themes: gender norms and decision-making roles, men’s perceptions of LARC side effects, and interactions with healthcare providers. These themes provide deeper insight into the patterns observed in the quantitative results and help explain the gap between high levels of service contact and limited male involvement.

Under the theme of gender norms and decision-making roles, the findings revealed that family planning is still widely perceived as a woman’s responsibility, which significantly limits male participation. This helps explain the quantitative result showing that although 96.4% of respondents had discussed contraception with a healthcare provider, a large proportion of men (77.3%) reported that they had never been directly involved in these discussions (χ² = 31.366, *p* < 0.001). Men described feeling excluded from decision-making processes during clinic visits, as illustrated by one participant who stated, “*When I go to the clinic*,* they assume it’s my wife’s decision. I also have concerns*,* but many men fear being judged or seen as less of a man if they talk about contraception*” (FGD, Man 5). These findings suggest that prevailing social expectations around masculinity discourage men from actively engaging in reproductive health matters.

The second theme, men’s perceptions of LARC side effects, highlighted persistent fears and misconceptions that act as barriers to involvement. While the quantitative findings showed no statistically significant differences regarding whether healthcare providers adequately addressed men’s concerns (χ² = 17.663, *p* = 0.344), the qualitative data revealed substantial variability in men’s experiences. Many participants expressed concerns related to infertility, reduced sexual performance, and long-term health effects, which were not always sufficiently addressed during consultations. A healthcare provider confirmed this, noting that “*Many men believe long-term methods cause permanent infertility or affect sexual performance. There is also stigma because family planning is seen as a woman’s domain*” (KII, Reproductive Health Officer 1). This variation helps explain the lack of statistical significance in the quantitative findings.

The third theme, interactions with healthcare providers, revealed a disconnect between access to services and meaningful male inclusion. Although most respondents had interacted with healthcare providers, these interactions were often directed primarily toward women, leaving men feeling sidelined. This aligns with the quantitative finding that, despite strong support for increased provider-led education (over 90%), the association was not statistically significant (χ² = 12.309, *p* = 0.421). Qualitative findings suggest that this reflects a shared recognition of unmet informational needs rather than differences between groups. Participants emphasized the importance of directly engaging men in family planning discussions, as illustrated by one respondent who stated, “*Healthcare providers need to speak directly to men. When we see other men involved*,* it feels more normal. It’s not just a woman’s responsibility*” (FGD, Woman 4).

Healthcare providers further highlighted practical strategies to improve male involvement, including promoting couple-based counseling, actively inviting men into consultations, and addressing misconceptions about LARC safety and reversibility. As one provider explained, “*We try to include both partners and explain that family planning is a shared responsibility. Creating a comfortable environment for men is essential*” (KII, Family Planning Coordinator 1). Overall, these findings demonstrate that male involvement in LARC decision-making is shaped by a complex interplay of social norms, knowledge gaps, and health system practices, underscoring the need for more inclusive and gender-responsive approaches to family planning.

**Table 4:** Multivariable Logistic Regression Analysis of Factors Associated with High Male Involvement in Long-Acting Reversible Contraceptive (LARC) decision-making among couples in Kampala, Uganda

Variable	Category	AOR	95% CI	*p*-value
Marital Status	Married	2.45	1.30–4.62	0.005*
	Single (Ref)	1	–	–
Education Level	Secondary	1.88	1.02–3.45	0.041*
	Diploma+	2.67	1.30–5.48	0.007*
	Primary/None (Ref)	1	–	–
Number of Children (Parity)	1–2 children	1.75	0.95–3.22	0.072
	3–4 children	2.31	1.25–4.27	0.008*
	≥ 5 children	1.9	0.80–4.50	0.14
	None (Ref)	1	–	–
Cultural Norms Influence	Yes	0.56	0.32–0.98	0.042*
	No (Ref)	1	–	–
Religious Influence	Yes	0.68	0.40–1.15	0.15
	No (Ref)	1	–	–
Awareness of LARC	Yes	3.1	0.40–24.2	0.28
	No (Ref)	1	–	–

The Table 4 above shows multivariable logistic regression analysis which was conducted to identify independent predictors of high male involvement in LARC decision-making. After adjusting for potential confounders, marital status, education level, and number of children remained significant predictors. Married participants were significantly more likely to be involved compared to single individuals (AOR = 2.45, *p* = 0.005). Higher education levels were also associated with increased involvement, particularly among those with diploma-level education or higher (AOR = 2.67, *p* = 0.007).

Participants with three to four children had higher odds of involvement (AOR = 2.31, *p* = 0.008), suggesting that experience with family size influences engagement in contraceptive decisions. Conversely, cultural norms negatively influenced male involvement (AOR = 0.56, *p* = 0.042), indicating that traditional beliefs may act as barriers.

These findings demonstrate that both socio-demographic and socio-cultural factors independently influence male participation in LARC decision-making.

## Discussion

This study contributes to the existing literature by providing context-specific insights from Kampala on the gap between awareness and actual male involvement in long-acting reversible contraceptive (LARC) decision-making [11]. First, although awareness of LARC methods was nearly universal (98.6%), and implants were the most recognized method (81.8%), joint decision-making remained low (38.7%), highlighting a critical disconnect between knowledge and practice. This finding adds to the literature by demonstrating that in the Kampala context, high awareness alone may not translate into shared decision-making, likely due to persistent gender norms and relational dynamics [7].

Second, the study highlights a distinct pattern of male involvement characterized by financial support but limited physical or communicative engagement. While 60.2% of men reportedly provided financial support, only 9.7% accompanied their partners to clinics, and only 17.4% reported frequent discussions about LARC. This suggests that in this setting, male involvement is often passive rather than active. Unlike previous studies that broadly describe male involvement, this study differentiates types of involvement, showing that financial contribution does not necessarily equate to meaningful participation in decision-making [13].

Third, the findings demonstrate that health system practices may unintentionally reinforce male exclusion, despite high levels of provider contact. Although 96.4% of respondents had interacted with healthcare providers, 77.3% reported that men were not directly involved in family planning discussions. This study adds to the literature by showing that provider–client interactions in Kampala remain largely woman-centered, which may limit opportunities for couple-based decision-making [4]. The qualitative findings further reinforce this, showing that men often feel sidelined during clinic visits.

Fourth, this study identifies socio-demographic and cultural factors associated with male involvement, including marital status, education level, and parity. Importantly, these findings should be interpreted as associations rather than causal relationships due to the cross-sectional design. Higher education was associated with increased male involvement (AOR = 2.67), suggesting that education may be linked to greater awareness and openness to shared decision-making, rather than directly causing it. Similarly, men with more children were more likely to be involved, which may reflect increased exposure to reproductive health decisions over time [5].

The findings suggest several targeted programmatic actions. First, the low level of male accompaniment to clinics (9.7%) and limited direct involvement by providers (77.3% not involved) indicate the need for male-friendly and couple-centered services, including actively inviting men to attend consultations and restructuring clinic environments to support joint counseling. Second, the persistence of misconceptions about LARC side effects, as highlighted in both quantitative (35.7%) and qualitative findings, supports the need for targeted health education interventions specifically designed for men, addressing fears related to infertility and sexual performance. Third, given that communication between partners remains low (only 17.4% reporting frequent discussions), community-based interventions that promote couple communication and male engagement, including peer education and male role models, may help normalize male participation. Finally, since healthcare providers were identified as the primary source of information (43.9%), strengthening provider training on inclusive communication and gender-sensitive counseling is essential to ensure that men are not unintentionally excluded during service delivery.

## Qualitative insights and integration with quantitative data

The survey results were supported by qualitative findings from focus groups and interviews, which highlighted key concerns regarding male participation in family planning, particularly for long-acting reversible contraceptives (LARCs). Many women reported that men were hesitant to engage because they lacked direct interaction with healthcare providers. As one participant explained, *“I believe my husband would feel more at ease if the doctor spoke to him directly about LARC methods.”* Despite the majority of women discussing contraception with providers, men were rarely involved. One woman noted, *“I’ve visited the clinic numerous times to talk about contraception*,* but my husband has never been involved.”*

Both men and women emphasized the need for joint education and counseling. A male participant said, *“If I had more information about contraception*,* I would take part more*,*”* while a woman added, *“If my husband understood the options*,* he would be more supportive.”* Nevertheless, 77.3% of participants reported that men were not included in consultations by healthcare providers.

Misconceptions about LARCs also limited male involvement. A healthcare provider observed, *“Many men are scared by the idea that these methods are not reversible.”* Despite these barriers, there is a growing recognition of shared responsibility. Participants expressed the importance of men being involved: *“It’s important that men are involved*,*”* said one woman, while a man remarked, *“It’s my responsibility to understand contraception.”*

Overall, male engagement remains minimal. Men often provide financial support but rarely accompany their partners to clinics, a finding consistent with survey results showing that male participation is largely limited to financial contributions rather than active involvement in decision-making.

## Limitations

This study has several limitations. First, it was conducted in a single urban facility in Kampala, which may limit the generalizability of the findings to rural settings or other regions. Second, the study relied predominantly on women’s reports to assess male involvement, which may not fully capture men’s perspectives or behaviors. Third, the use of self-reported data introduces the possibility of social desirability bias, particularly in responses related to gender roles, religion, and support for family planning, where participants may have provided socially acceptable answers rather than reflecting actual practices. Additionally, the cross-sectional design limits the ability to establish causal relationships between variables, and findings should therefore be interpreted as associations.

## Conclusion

This study provides important new information about the variables influencing men’s participation in long-acting reversible contraceptive (LARC) decision-making in Kampala, Uganda. The results show that male involvement is primarily restricted to offering financial assistance, despite the fact that there is a high level of awareness about LARC methods and many people think using contraceptives improves relationships. This disparity highlights the necessity of defining male involvement more broadly to encompass active decision-making, practical help, and emotional support in addition to monetary contributions. Income, the number of children, education, marital status, and polygamous partnerships all have a significant impact on men’s participation in LARC choices. Generally speaking, family planning is more common among males in stable marriages who are better educated, have more children, and earn more money. However, active engagement is frequently limited by misconceptions regarding partner preferences, side effects, and traditional gender roles. Religious and cultural convictions can provide serious obstacles to male participation. These difficulties, along with the lack of candid communication between partners over family planning, underscore the need of initiatives that encourage open communication and tackle societal and cultural norms that restrict men’s use of contraceptives.

## Supplementary Information

Below is the link to the electronic supplementary material.

Supplementary Material 1

## Data Availability

The datasets generated and/or analyzed during the current study are available from the corresponding author upon reasonable request. Due to the sensitive nature of the information collected, all data have been anonymized to protect participants’ identities.
